# Microbial protein: future sustainable food supply route with low environmental footprint

**DOI:** 10.1111/1751-7915.12369

**Published:** 2016-07-08

**Authors:** Silvio Matassa, Nico Boon, Ilje Pikaar, Willy Verstraete

**Affiliations:** ^1^ Center of Microbial Ecology and Technology (CMET)Ghent UniversityCoupure Links 6539000GentBelgium; ^2^ Avecom NVIndustrieweg 122P9032WondelgemBelgium; ^3^ The School of Civil EngineeringThe University of QueenslandSt. LuciaQLD4072Australia; ^4^ KWR Watercycle Research InstitutePost Box 10723430BB NieuwegeinThe Netherlands

## Abstract

Microbial biotechnology has a long history of producing feeds and foods. The key feature of today's market economy is that protein production by conventional agriculture based food supply chains is becoming a major issue in terms of global environmental pollution such as diffuse nutrient and greenhouse gas emissions, land use and water footprint. Time has come to re‐assess the current potentials of producing protein‐rich feed or food additives in the form of algae, yeasts, fungi and plain bacterial cellular biomass, producible with a lower environmental footprint compared with other plant or animal‐based alternatives. A major driver is the need to no longer disintegrate but rather upgrade a variety of low‐value organic and inorganic side streams in our current non‐cyclic economy. In this context, microbial bioconversions of such valuable matters to nutritive microbial cells and cell components are a powerful asset. The worldwide market of animal protein is of the order of several hundred million tons per year, that of plant protein several billion tons of protein per year; hence, the expansion of the production of microbial protein does not pose disruptive challenges towards the process of the latter. Besides protein as nutritive compounds, also other cellular components such as lipids (single cell oil), polyhydroxybuthyrate, exopolymeric saccharides, carotenoids, ectorines, (pro)vitamins and essential amino acids can be of value for the growing domain of novel nutrition. In order for microbial protein as feed or food to become a major and sustainable alternative, addressing the challenges of creating awareness and achieving public and broader regulatory acceptance are real and need to be addressed with care and expedience.

## Introduction

From the times when our ancestors decided to settle, growing crops and domesticating animals became consolidated practices allowing constant feed and food production. As human civilization proceeded, new strategies of securing food supply have continuously been discovered, consolidated and improved. The major driver of such process was the need to provide resilience towards the changing elements of nature, continuously threatening food supply (Berglund, 2003).

The current anthropogenic pressure on earth's finite resources and the concomitant dynamics of climate change, generate serious concerns about the resilience of the contemporary agricultural feed/food chains (Godfray *et al*., 2010). In view of the still growing world population towards 10 billion in 2050 (Ezeh *et al*., 2012), it has been calculated that the world will need to produce about 70% more food calories than in 2006 (Ranganathan, 2013). Therefore, there is a need to find reliable alternative solutions, able to strengthen future food security while minimizing the impact on the global sustainability.

Microorganisms have always been central in basic food processing techniques, for instance converting fibres into edible food when fermenting dough to produce bread, or milk into cheese, allowing its long‐term preservation (Caplice and Fitzgerald, 1999). They have been often used as direct food source, as it is the case for yeast or algae. The latter, together with bacteria, constitute the microbial actors involved in processing food. They can also be used directly as feed or food source (Anupama and Ravindra, 2000). The term ‘microbe’ is used here in the broad connotation of bacteria, fungi, yeast and algae.

In the early 1960s, when public awareness grew in respect to the impeding global demographic boom, the need to search for alternatives to sustainably feed a growing population corresponded in major efforts to develop alternative feed and food sources (Goldberg, 1985). Several attempts were made to develop and bring to practice the production of high‐quality protein additives from microorganisms, known as microbial protein (MP), or single cell protein (SCP), mainly by using abundant and low cost hydrocarbon substrates such as methanol and methane (Goldberg, 1985). The Imperial Chemical Industries (ICI) were the first to bring to full scale production and commercialization a MP product called Pruteen^®^, produced from methanol oxidation by means of *Methylophilus methylotrphus* (Westlake, 1986). Besides industrially developed hydrocarbon‐based MP, researchers investigated a whole range of other possibilities to produce MP, including the use of natural or artificial light, molecular hydrogen and many different organic substrates such as by‐products from the sugar industry as well as other food processing residues or even food wastes (Anupama and Ravindra, 2000). Despite being well accepted and successful in many feed trials with livestock, the actual and definitive breakthrough of MP in the animal feed market was hampered by the low prices achieved by more conventional protein sources such as soybean and fishmeal in the late 1970s as well as the fairly underdeveloped state of fermentation technology. Concomitantly, the rising oil prices in the subsequent decades led to the end of the ICI enterprise because of the relatively high costs of MP production and the consequent competitive disadvantage towards other cheaper more ‘natural’ alternatives (Øverland *et al*., 2010).

In recent years, however, research and development around MP is regaining momentum both in the scientific and industrial domains. The steep increase in the prices of fishmeal (from about $500 per ton in the 1990s to $1500 to $2500 in recent years), together with the environmental pressure of soybean production on land and water use in the tropical areas of the globe justify the re‐examination of the microbial alternative (Kupferschmidt, 2015).

In the present article, we align the possibilities offered as well as the challenges to be faced by the use of MP production as a biotechnological tool to help securing nutritive protein supply in the years to come. Threatened by forthcoming population growth, climate change and agricultural unsustainability, mankind must seek, once more, new forms of adaptation to safeguard itself.

## Microbial protein: feed, food and further

### MP as feed

The main driver leading to the renaissance of MP as a source of feed is indubitably the aquaculture sector. Fish farming currently provides about 50% of world's fish food supply, and it is projected to grow further, becoming a key sector in the supply of high‐quality protein for the global population. In this context, scientific research and the industrial applications have found in MP a powerful ally. Aquaculture accounts nowadays for more than 73% of the global fishmeal consumption, with wild fish capture clearly unable to provide enough high‐quality feed for such a fast growing sector (The World Bank, 2013). Production of MP from natural gas has recently received a great deal of attention, with innovative fermentation processes allowing high volumetric productivities (3–4 kg MP dry matter (DM) per m^3^ reactor volume per hour) by continuous cultures of *Methylococcus capsulatus*, marketed under the name of FeedKind^®^ (Unibio, 2016). The latter level of productivity has a physical footprint which is a factor 1000, or more, smaller than any conventional vegetable protein production system (Matassa *et al*., 2015a). Besides achieving feasible industrial scale production and costs competitiveness with fishmeal, the final MP product is comparable to fishmeal in terms of essential amino acid profile and overall nutritive value (Øverland *et al*., 2010). Being tested in numerous feed trials with different fish species, resulting in promising perspectives, full‐scale production is currently ongoing, with a production of up to 80 000 ton DM/year foreseen in the near future (see Table 2).

In addition to aquaculture, the MP product has also been successfully tested in feed trials with terrestrial animals including major livestock like ruminants, pigs and chickens, broadening its potential market applications (Øverland *et al*., 2010). In this case though, the relatively low price of soybean meal and the abundant and well‐established use as main protein additive in livestock production of the latter, still counteract the application of natural gas‐based MP as replacement of substantial percentages of feed composed by fishmeal.

An alternative route to produce MP consists of recovering valuable nutrients from various side streams of the food industry, for instance feed and food processing water (Lee *et al*., 2015). In this case, the use of heterotrophic microorganisms such as yeast and bacteria allows to convert the organic carbon and the nutrients (N, P) in the waste or processing waters into MP (Anupama and Ravindra, 2000). Microbial protein produced along this line might constitute a valuable and competitive route to produce a substitute for soy protein for animal feed. Indeed it should be possible to generate such MP at costs which take into account the revenue from the avoidance of the treatment of the mineral nutrients (N, P) present in side (waste) streams. As a matter of fact, dissipation of reactive nitrogen back to atmosphere as dinitrogen gas by means of the conventional nitrification–denitrification pathway comes to a cost of about 2–3 Euro per kg nitrogen‐N, while capture of phosphorous in the wastewater line costs of the order of 7 Euro per kg P (Levlin and Hultman, 2003). Note that the market value of proteinaceous nitrogen from vegetable sources is at the current price of some 1.1–1.6 Euro per kg dry weight protein (see Table 1) corresponding to some 6 Euro per kg proteinaceous N. When this microbial proteinaceous N is converted to high‐value animal protein, one can attain an end‐value of the same order and even up to 14 Euro per kg dry weight protein, in case of fish.

**Table 1:** Production volumes and price of various animal and vegetable protein sources

Protein source	Production volume (Mton DM/y)	Farm gate price ($/kg DM)	Average protein content (% DW)	Price per unit protein ($/kg protein DM)	Ref
Animal					
Fish	66.7	2.07	15–20	10–14	Waite *et al*. (2014)
Pork	108.5	1.54	20	7.7	Waite *et al*. (2014)
Chicken	92.7	1.43	31	4.6	Waite *et al*. (2014)
Beef	62.7	2.70	25	10.8	Waite *et al*. (2014)
Vegetable					
Soybean	320.2	0.37	35	1.1	Indexmundi (2016a); USDA (2015)
Wheat	712.7	0.19	12	1.6	FAO, 2015; Indexmundi (2016b)

An example of the implementation of such approach to food processing water is the study published by Lee *et al*. (2015), where the effluent of a brewery is used as feedstock for the production of SCP‐MP. The latter study relates to a technology implemented by Nutrinsic, dealing with a production volume of 5000 ton DM/year from a brewery effluent (see Table 2). Also, at present in Belgium, a first full‐scale MP production installation is under construction dealing with the upgrading of potato process waters. It should be in production by 2016 at the level of 5000 ton MP per year (Valpromic NV, pers. comm.). Yet, for the latter bacterial‐based MP products, there are so far no clear cut data in terms of their putative market size or market values. Nevertheless, the sector is attracting growing interests from investors dealing with novel aspects of the cyclic economy (Nutrinsic, 2014).

**Table 2:** Overview of current production volumes and market sizes for different microbial protein. Hyphens indicate that values were not available

Organisms	Production volume (ton DM/y)	Production costs (Euro/kg DM)	Global market value (Billion Euro)	Yearly growth (% per year)	Remarks	Ref
Yeast	3 000 000	–	9.2	7.9	Mostly commercialized as baker's yeast and for ethanol fermentation. Global market value projected to 2019	Kellershohn and Russell (2015)
Algae (microalgae)	9000	4–25	2.4	10	Besides feed and food, derivatives are also used	Enzing *et al*. (2014)
Mycoprotein (Quorn^®^)	25 000	–	0.214	20	Investments for a plant of 22000 tons per year were done in 2015	Beer (2015)
Bacteria (Profloc^®^)	5000	1–1.1	–	–		Nutrinsic (2015)
Bacteria (FeedKind^®^)	80 000	–	–	–	Commercial production foreseen on 2016	Byrne (2016)
Valpromic	5000	–	–	–		Personal communication

### MP as food

**Figure 1:**
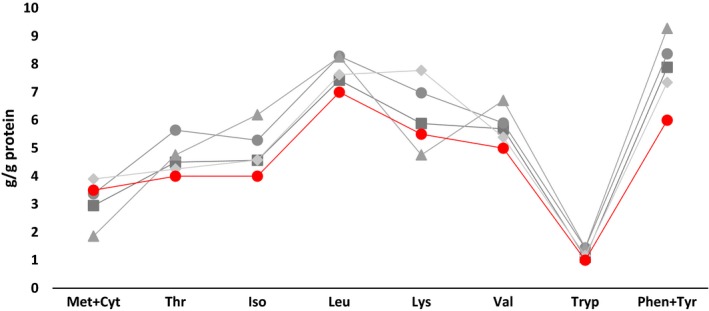
Essential amino acid scoring pattern of microbial protein from bacteria (Pseudomonas/Methylophilus spp.) (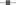), yeast (Candida spp.) (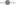), algae (Spirulina maxima (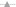), compared with the high‐quality animal protein from fishmeal (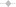) as well as to the FAO/WHO standard (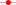) for amino acid scoring pattern for human nutrition. Source: Harper (1981); Tacon (1987)

Microbial protein is an alternative source of high‐quality protein able to replace animal protein like fishmeal in livestock nutrition and aquaculture. Going one step higher in the food chain, MP is meeting the FAO/WHO requirements in terms of essential amino acid scoring pattern for human nutrition (Fig. 1) and therefore, also humans could benefit greatly from the use of MP directly as food.

Algae are reported to have supported the life of ancient populations living close to the sea for millennia, providing a constant source of protein and vitamins. Algae and microalgae are currently used as food and food supplements in food industry (Anupama and Ravindra, 2000; Becker, 2007), with a global production achieving 9000 ton DM/year (see Table 2) with a market value estimated about 2.4 billion Euro with a projected yearly growth of 10%.

Yeast, being at the base of food processing since the first bread was baked, or grapes fermented, can also be used as direct food source, as it was the case e.g. with the massive campaign of yeast production and supply, first to the army, then to the whole population during World War II (Khachatourians and Arora, 2002). Currently, yeast is a major player in the microbial derived production of products for food as well as for other applications. Baker's yeast and alcohols fermentation are the two main processes employing yeast, with a projected global market value for 2019 of up to 9.2 billion Euro and an annual growth forecast of 7.9% (see Table 2).

Fungi are also a suitable alternative and have also made their way as human food. Quorn^™^ is the most successful example of the so called mycoprotein, which is commercialized and sold in some 15 countries worldwide (Wiebe, 2004). Mycoproteins are particularly suited to reproduce the taste and consistency of meat; this explains their success as alternative to conventional animal‐based products. Currently, mycoprotein production supporting Quorn^™^ products manufacturing amounts to 25 000 ton DM/year, with a global market value of about 214 million Euro, prospected to grow with 20% annually in the coming years.

### Added value applications

Besides being rich in nutritive protein, microorganisms offer the possibility of producing a broad variety of added‐value products, suitable for both animal and human nutrition (Vandamme and Revuelta, 2016). Table 3 summarizes the average amount of protein producible by algae, fungi and bacteria, as well as other possible added‐value products already investigated or produced from microorganisms.

**Table 3:** Overview of different microorganism for MP and added‐value product formation

Microorganism	Average crude protein content (% CDW)	Nutritional value	Added value by‐products (% CDW)	Remarks	Ref.
Algae	40–60	Compares favourably to egg, soy and what protein. Cell wall digestibility is an issue	Microbial oil (50–70%) Carbohydrates (up to 70%) Vitamins …	Triacylglycerides (TAG) can replace partly vegetable oils in food products. Poly unsaturated fatty acids (PUFA) are of interest for health applications	Draaisma *et al*. (2013); Harun *et al*. (2010)
Fungi (Filamentous and Yeast)	30–70	Amino acids and digestibility of mycoprotein is similar to egg and milk	Carbohydrates Pullulan Xylitol Astaxanthin …	High unsaturated/saturated fatty acids and low fat content makes them highly suitable for human nutrition	Thrane (2007)
Bacteria	50–83	Amino acids and digestibility is similar to those of fishmeal	Internal storage polymers (PHB) Ectoine Lipids Extracellular polysaccharides Growth media and vitamins …		Strong *et al*. (2015)

Certain microalgae and cyanobacteria are primary producers of microbial oil, suitable as substitutes for vegetable oil in food supplements. Particularly, the high concentration of fatty acids can replace fatty acids otherwise derived from rape seed, soy, sunflower oil and palm oil. The purification of omega‐3 fatty acid can offer even higher value applications, e.g. for clinical purposes, eicosapentanoic acid and decosahexaenoic acid, normally obtained from fish oil, can be also concentrated and purified from naturally omega‐3‐accumulating microalgae. Vitamins such as vitamin B12 and provitamin A are also important high‐value products obtainable from edible algae, conferring additional nutritional benefits in livestock production. Carbohydrates, which can be accumulated up to 70% of the cell dry weight by many algal species are also of nutritional value, but the major research effort so far was directed towards the use of algae for biofuel, biogas or biohydrogen generation (Jones and Mayfield, 2012; Draaisma *et al*., 2013).

Fungi, mainly yeast‐like fungi are the main agents involved in the saccharification of fibres from corn, as well as fermentation of other organic substrates. While processing corn fibre with yeast like *Aureobasidium*, xylose, arabinose and glucose can be produced at different relative concentrations depending on the pre‐treatment of the fibres feedstock. The sugars can then be further fermented in bioethanol, xylitol and pullulan. Xylitol and pullulan find particularly application as food additive for their specific property of flavour‐enhancing and binding agents. Besides sugars and sugar‐derivatives, yeasts like *Phaffa rhodozyma* (now Xanthophyllomyces rodochrous) can be used to produce valuable carotenoid pigments like astaxanthin, mainly used in aquaculture as feed supplement for salmon (Leathers, 2003).

Bacteria are a versatile group of microorganisms able to produce a large array of added‐value bio‐products. Biopolymers such as polyhydroxyalkanes (PHA) are named to be biological alternatives to petroleum‐based chemicals to produce plastics. Yet, so far large‐scale applications are to the best of our knowledge, not industrially established. Recently, other applications for PHA/polyhydroxybuthyrate (PHB) such as in the medical field are emerging. Of interest for aquaculture is the ongoing research on the prebiotic effects of PHB when used as feed supplement, offering an interesting alternative to antibiotics (Defoirdt *et al*., 2007; De Schryver *et al*., 2010). Another interesting niche product which can be derived from bacteria is osmo‐protectants such as glutamate and ectoine (Lentzen and Schwarz, 2006). The latter is a high‐value cyclic imino acid used in cosmetic formulation, but which has found application also in aquaculture as highly active protectant against oxidative stress. Bacteria can also produce relevant amounts of lipids, commonly employed in biofuel production. High‐quality membrane‐derived lipids can also be employed as human health supplement, being already tested as effective in reducing plasma cholesterol during animal tests (Strong *et al*., 2015).

## Forthcoming challenges

**Figure 2:**
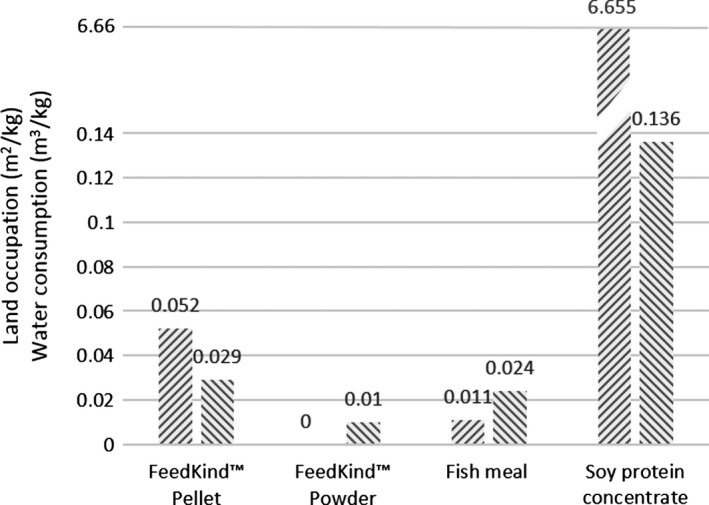
Land (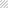) and freshwater (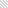) requirements of MP compared with fishmeal and soy protein concentrate. The values are normalized to the protein content of each product. Source: Cumberlege *et al*. (2016)

The extensive use of MP products as partial replacement of conventional protein feed additives such as soybean and fishmeal can offer the opportunity of decreasing part of the environmental pressure that these products exert on land and water use. A recent report of the British Carbon Trust evaluated the environmental impact of FeedKind^®^ protein, a bacterial MP feed additive produced from natural gas (see *MP as feed*). The report evaluated two FeedKind^®^ commercial products in terms of greenhouse gas, land and water use, comparing them with soybean and fishmeal (Cumberlege *et al*., 2016). In terms of freshwater consumption, the report shows an average value of about 29 m^3^ per ton MP produced. A more detailed analysis shows that this 29 m^3^ is for about 80% determined by the vegetable oil used as binding agent to produce a MP‐pelletized product. If the latter major contribution is excluded by producing a simple straightforward protein powder, the actual freshwater requirement comes down to the order of 1 m^3^ per ton MP. From Fig. 2, it can be derived that this value about water foot print is about 20 and 140 times lower than fishmeal and soybean meal respectively.

The same trend is observable for the required land. The value of 52 m^2^ per ton MP is in fact due to vegetable oil for the production of the pelletized form, whereas no arable land is required in case of the powdered MP. Compared with the 6655 m^2^ land per ton protein required for the production of soybean meal concentrate, the value of quasi zero land foot print reveals how the land footprint of MP production is a major benefit in respect to conventional agricultural‐based protein production. Fishmeal of course requires minimal amount of land for its processing, yet the dramatic impact of wild fish capture on ocean ecosystems is well known and documented (Pauly *et al*., 2005).

Finally, the above‐mentioned report also analyses the carbon footprint of FeedKind^®^. The value of 5.8 ton CO_2eq_/ton MP is mainly due to the natural gas necessary for the metabolism of the bacteria involved in the biological fermentation process. This value would be as low as 1.7 ton CO_2eq_/ton MP in case biogas and renewable energy is used in place of fossil fuels to power the reactor‐based production and downstream processing of the final MP product. For fishmeal and soybean meal concentrate, the report indicates values of 2.6 and 0.8 ton CO_2eq_/ton protein respectively. Nevertheless, if the spared agricultural land in case of MP production would be accounted for its recovered carbon capture potential, the overall benefit in avoided carbon emissions from MP production could possibly be much higher.

In this context, an interesting alternative to natural gas‐based MP is represented by autotrophic microorganisms such as algae or hydrogen‐oxidizing bacteria. If algae offer the great advantage of being able to use sun light to fix carbon dioxide into biomass, the main drawbacks of such process are the high land footprint required together with the technical challenges of downstream processing of poorly concentrated algal biomass (Majid *et al*., 2014). On the other hand, the hydrogen gas needed to fix carbon dioxide into bacterial biomass by means of hydrogen‐oxidizing bacteria is more expensive resource, but the land footprint and the biomass concentrations achievable with modern fermentation technologies outscore those of the algal platform. In case the production of the latter is connected to hydrogen generated by means of renewable energies (solar, wind, etc.), this allows an elegant platform for MP production and concomitant carbon dioxide capture (Matassa *et al*., 2015b).

To the scientific community, it is evident how a dedicated industrial production of MP can represent a key biotechnological tool to curb down the environmental impact of the current feed and food chain assuring the necessary amounts of nutritive protein for mankind. Clearly, significant efforts are warranted to bring this to practice at relevant scales. A key feature is to deal with the aspects of public awareness. At present, the mere economic market rules justify the application of MP in feed for livestock only in some niche applications such as aquaculture. Yet, if the externalized environmental costs of the current feed/food production system would be taken into account and made clear to the broader public (including decision makers in political institutions), the MP route would result in a more rational alternative, able to offer immediate advantages in terms of water and land use, with direct consequences on increased carbon capture potential of ecosystems restored by better agricultural land use (Galloway and Leach, 2016). An important aspect, in this sense, relates to nutrients flows, and principally the excessive input in our biosphere of reactive nitrogen species (NH_4_^+^, NO_2_^−^, NO_3_^−^) produced by fixing atmospheric N_2_ gas by means of the Haber–Bosch process. Compared with the proposed sustainable boundary of 35 Mton N_2_ fixed per year, the current 121 Mton actually converted into reactive nitrogen surpass the sustainability boundary of almost 3.5 times (Rockstrom *et al*., 2009). Moreover, if the economic benefit in agricultural production ranges between 20 and 80 billion Euro per year, the annual costs (including damages to both environment and human health) of N pollution by agriculture have been estimated in the range of 35–230 billion Euro per year (Van Grinsven *et al*., 2013). It has been recently demonstrated how the high nitrogen inefficiency of the soil–plant system could be mitigated by MP production (Matassa *et al*., 2015a), decreasing significantly the impact of eutrophication, nitrous oxide emissions and ecosystems disturbance due to unbalanced anthropogenic nitrogen inputs.

Besides the awareness of the overall environmental benefit of MP production for feed and food, the development of higher value by‐products will allow boosting and bolstering the MP biotech platform. Thus, a more powerful penetration into the market of microbial‐based product as replacement of chemically derived ones, as discussed above, will be possible. This will play in favour of establishing a public mindset more open and prone to acceptance towards microbial derived products.

Obviously, barriers must be overcome in order to allow a widespread adoption of the MP biotechnology. Besides the official legal recognition of some MP products as feed and food (Øverland *et al*., 2010), further openings are warranted in terms of used carbon and nutrient sources recovery and their up‐cycling into edible MP products as part of the cyclic economy. This will impact drastically on how efficiently our current society makes use of its precious primary resources.

## Conclusions

- Microbial protein qualifies as an excellent source of nutritive proteins, but other cellular components can also be of increasing importance, driving new developments for microbial‐based by‐products. In the context of the need to generate new biotechnological processes and products, this line of research and development, particularly in countries where new opportunities are warranted, should be explored with great care (Timmis *et al*., 2014).
- The current conventional agricultural based supply route for nutritive animal proteins have a high environmental impact; they should be reconsidered and their externalized environmental costs should be assessed and benchmarked to those of MP.
- Upgrading various valuable nutrients (N, P) and nutritive resources (organic carbon), the production of heterotrophic microbes as food and feed certainly has gaining renewed industrial interest, particularly in the context of the cyclic economy.
- The overall transition of the public mindset towards widespread acceptance and appreciation of MP as main supply route for feed and food needs to be prepared in the near future with care and foresight particularly in terms of quality and regulatory issues.

## References

[mbt212369-bib-0001] Anupama , and Ravindra, P. (2000) Value‐added food: single cell protein. Biotechnol Adv 18: 459–479. 14538097 10.1016/s0734-9750(00)00045-8

[mbt212369-bib-0002] Becker, E.W. (2007) Micro‐algae as a source of protein. Biotechnol Adv 25: 207–210. 17196357 10.1016/j.biotechadv.2006.11.002

[mbt212369-bib-0003] Beer, E. (2015) “No success model” for new protein ingredients [WWW Document]. URL http://www.foodnavigator.com/Market-Trends/No-success-model-for-new-protein-ingredients

[mbt212369-bib-0004] Berglund, E. (2003) Human impact and climate changes—synchronous events and a causal link? Quat. Int. 105: 7–12.

[mbt212369-bib-0005] Byrne, J. (2016) Calysta says gas to fishmeal replacement protein on path to commercialization [WWW Document]. URL http://www.feednavigator.com/R-D/Calysta-says-gas-to-fishmeal-replacement-protein-on-path-to-commercialization

[mbt212369-bib-0006] Caplice, E. , and Fitzgerald, G.F. (1999) Food fermentations: role of microorganisms in food production and preservation. Int J Food Microbiol 50: 131–149. doi:10.1016/S0168‐1605(99)00082‐3. 10488849 10.1016/s0168-1605(99)00082-3

[mbt212369-bib-0007] Cumberlege, T. , Blenkinsopp, T. and Clark, J. (2016) Assessment of environmental impact of FeedKind protein Carbon Trust, URL https://www.carbontrust.com/media/672719/calysta-feedkind.pdf.

[mbt212369-bib-0008] De Schryver, P. , Sinha, A.K. , Kunwar, P.S. , Baruah, K. , Verstraete, W. , Boon, N. , *et al* (2010) Poly‐beta‐hydroxybutyrate (PHB) increases growth performance and intestinal bacterial range‐weighted richness in juvenile European sea bass, Dicentrarchus labrax. Appl Microbiol Biotechnol 86: 1535–1541. doi:10.1007/s00253‐009‐2414‐9. 20094715 10.1007/s00253-009-2414-9

[mbt212369-bib-0009] Defoirdt, T. , Boon, N. , Sorgeloos, P. , Verstraete, W. , and Bossier, P. (2007) Alternatives to antibiotics to control bacterial infections: luminescent vibriosis in aquaculture as an example. Trends Biotechnol 25: 472–479. doi:10.1016/j.tibtech.2007.08.001. 17719667 10.1016/j.tibtech.2007.08.001

[mbt212369-bib-0010] Draaisma, R.B. , Wijffels, R.H. , Slegers, P.M.E. , Brentner, L.B. , Roy, A. , and Barbosa, M.J. (2013) Food commodities from microalgae. Curr Opin Biotechnol 24: 169–177. doi:10.1016/j.copbio.2012.09.012. 23084075 10.1016/j.copbio.2012.09.012

[mbt212369-bib-0011] Enzing, C. , Ploeg, M. , Barbosa, M. and Sijtsma, L. (2014) Microalgae‐based products for the food and feed sector: an outlook for Europe. doi:10.2791/3339.

[mbt212369-bib-0012] Ezeh, A.C. , Bongaarts, J. , and Mberu, B. (2012) Global population trends and policy options. Lancet 380: 142–148. doi:10.1016/S0140‐6736(12)60696‐5. 22784532 10.1016/S0140-6736(12)60696-5

[mbt212369-bib-0013] FAO (2015) World food situation [WWW Document]. URL http://www.fao.org/worldfoodsituation/csdb/en/

[mbt212369-bib-0014] Galloway, J.N. , and Leach, A.M. (2016) Sustainability: your feet's too big. Nat Geosci 9: 97–98.

[mbt212369-bib-0015] Godfray, H.C.J. , Beddington, J.R. , Crute, I.R. , Haddad, L. , Lawrence, D. , Muir, J.F. , *et al* (2010) Food security: the challenge of feeding 9 billion people. Science 327: 812–818. 20110467 10.1126/science.1185383

[mbt212369-bib-0016] Goldberg, I. (1985) Single Cell Protein, Biotechnology Monographs. Springer‐Verlag, Berlin.

[mbt212369-bib-0017] Harper, A. (1981) Amino Acid Scoring Patterns [WWW Document]. URL http://www.fao.org/3/contents/aa7e1ca5-4634-51bf-a465-5bf12d5cec2d/M3013E00.HTM

[mbt212369-bib-0018] Harun, R. , Singh, M. , Forde, G.M. , and Danquah, M.K. (2010) Bioprocess engineering of microalgae to produce a variety of consumer products. Renew Sustain Energy Rev 14: 1037–1047. doi:10.1016/j.rser.2009.11.004.

[mbt212369-bib-0019] Indexmundi (2016a) Soybeans [WWW Document]. URL http://www.indexmundi.com/commodities/?commodity=soybeans.

[mbt212369-bib-0020] Indexmundi (2016b) Wheat [WWW Document]. URL http://www.indexmundi.com/commodities/?commodity=wheat.

[mbt212369-bib-0021] Jones, C.S. , and Mayfield, S.P. (2012) Algae biofuels: versatility for the future of bioenergy. Curr Opin Biotechnol 23: 346–351. doi: 10.1016/j.copbio.2011.10.013. 22104720 10.1016/j.copbio.2011.10.013

[mbt212369-bib-0022] Kellershohn, J. and Russell, I. (2015) Yeast biotechnology In Advances in Food Biotechnology. John Wiley & Sons, pp. 303–310. doi:10.1002/9781118864463.ch18.

[mbt212369-bib-0023] Khachatourians, G.G. and Arora, D.K. (2002) Applied Mycology and Biotechnology, Volume 2. Amsterdam: Agriculture and Food Production.

[mbt212369-bib-0024] Kupferschmidt, K. (2015) Why insects could be the ideal animal feed [WWW Document]. Science. doi:10.1126/science.aad4709.

[mbt212369-bib-0025] Leathers, T.D. (2003) Bioconversions of maize residues to value‐added coproducts using yeast‐like fungi. FEMS Yeast Res 3: 133–140. doi:10.1016/S1567‐1356(03)00003‐5. 12702445 10.1016/S1567-1356(03)00003-5

[mbt212369-bib-0026] Lee, J.Z. , Logan, A. , Terry, S. , and Spear, J.R. (2015) Microbial response to single‐cell protein production and brewery wastewater treatment. Microb Biotechnol 8: 65–76. doi:10.1111/1751‐7915.12128. 24837420 10.1111/1751-7915.12128 PMC4321374

[mbt212369-bib-0027] Lentzen, G. , and Schwarz, T. (2006) Extremolytes: natural compounds from extremophiles for versatile applications. Appl Microbiol Biotechnol 72: 623–634. doi:10.1007/s00253‐006‐0553‐9. 16957893 10.1007/s00253-006-0553-9

[mbt212369-bib-0028] Levlin, E. and Hultman, B. (2003) Phosphorus recovery from phosphate rich side‐streams in wastewater treatment plants. In Polish Swedish Seminar, Gdansk March. pp. 47–56.

[mbt212369-bib-0029] Majid, M. , Shafqat, S. , Inam, H. , Hashmi, U. and Kazi, A.G. (2014) Biomass and Bioenergy: Processing and Properties. Hakeem R.K., Jawaid M. and Rashid U. (eds). Cham, Switzerland: Springer International Publishing, pp. 207–224. doi:10.1007/978‐3‐319‐07641‐6_13.

[mbt212369-bib-0030] Matassa, S. , Batstone, D.J. , Huelsen, T. , Schnoor, J.L. , and Verstraete, W. (2015a) Can direct conversion of used nitrogen to new feed and protein help feed the world? Environ Sci Technol 49: 5247–5254. doi:10.1021/es505432w. 25816205 10.1021/es505432w

[mbt212369-bib-0031] Matassa, S. , Boon, N. , and Verstraete, W. (2015b) Resource recovery from used water: the manufacturing abilities of hydrogen‐oxidizing bacteria. Water Res 68: 467–478. doi:10.1016/j.watres.2014.10.028. 25462753 10.1016/j.watres.2014.10.028

[mbt212369-bib-0032] Nutrinsic (2014) Nutrinsic Corporation Raises $12 [WWW Document]. URL http://www.marketwired.com/press-release/nutrinsic-corporation-raises-127-million-to-fuel-growth-1938044.htm

[mbt212369-bib-0033] Nutrinsic (2015) Nutrinsic Announces Grand Opening of First US ProFloc(TM) Facility. [WWW Document]. URL http://www.marketwired.com/press-release/-2020797.htm

[mbt212369-bib-0034] Øverland, M. , Tauson, A.‐H. , Shearer, K. , and Skrede, A. (2010) Evaluation of methane‐utilising bacteria products as feed ingredients for monogastric animals. Arch. Anim. Nutr. 64: 171–189. doi:10.1080/17450391003691534. 20578647 10.1080/17450391003691534

[mbt212369-bib-0035] Pauly, D. , Watson, R. , and Alder, J. (2005) Global trends in world fisheries: impacts on marine ecosystems and food security. Philos Trans R Soc Lond B Biol Sci 360: 5–12. 15713585 10.1098/rstb.2004.1574 PMC1636108

[mbt212369-bib-0036] Ranganathan, J. (2013) The Global Food Challenge Explained in 18 Graphics [WWW Document]. World Resour. Inst. URL http://www.wri.org/blog/2013/12/global-food-challenge-explained-18-graphics

[mbt212369-bib-0037] Rockstrom, J. , Steffen, W. , Noone, K. , Persson, A. , Chapin, F.S. , Lambin, E.F. , *et al* (2009) A safe operating space for humanity. Nature 461: 472–475. 19779433 10.1038/461472a

[mbt212369-bib-0038] Strong, P.J. , Xie, S. , and Clarke, W.P. (2015) Methane as a resource: can the methanotrophs add value? Environ Sci Technol 49: 4001–4018. doi:10.1021/es504242n. 25723373 10.1021/es504242n

[mbt212369-bib-0039] Tacon, G.J.A. (1987) The nutrition and feeding of farmed fish and shrimp ‐ a training manual 2. Nutrient sources and composition. FAO URL http://www.fao.org/3/contents/d66b3e1f-c059-50fa-9ba2-717e9940b7f1/AB470E00.htm

[mbt212369-bib-0040] The World Bank (2013) FISH TO 2030 Prospects for Fisheries and Aquaculture. doi:83177‐GLB.

[mbt212369-bib-0041] Thrane, U. (2007) Fungal protein for food In Food Mycology, Mycology. CRC Press, pp. 353–360. doi:10.1201/9781420020984.ch18.

[mbt212369-bib-0042] Timmis, K. , de Lorenzo, V. , Verstraete, W. , Garcia, J.L. , Ramos, J.L. , Santos, H. , *et al* (2014) Pipelines for New Chemicals: a strategy to create new value chains and stimulate innovation‐based economic revival in Southern European countries. Environ Microbiol 16: 9–18. doi:10.1111/1462‐2920.12337. 24387039 10.1111/1462-2920.12337

[mbt212369-bib-0043] Unibio (2016) Introduction _ Unibio [WWW Document]. URL http://www.unibio.dk/technology/introduction.

[mbt212369-bib-0044] USDA (2015) World agricultural supply and demand estimates. United States Dep. Agric. doi:WASDE‐525.

[mbt212369-bib-0045] Van Grinsven, H.J.M. , Holland, M. , Jacobsen, B.H. , Klimont, Z. , Sutton, M. , and Willems, W.J. (2013) Costs and benefits of nitrogen for europe and implications for mitigation. Environ Sci Technol 47: 3571–3579. 23473305 10.1021/es303804g

[mbt212369-bib-0046] Vandamme E.J. and Revuelta J.L. (eds.) (2016) Industrial Biotechnology of Vitamins, Biopigments, and Antioxidants. Weinheim, Germany: Wiley‐VCH, pp. 560.

[mbt212369-bib-0047] Waite, R. , Beveridge, M. , Brummett, R.E. , Castine, S. , Chaiyawannakarn, N. , Kaushik, S. , *et al* (2014) Improving productivity and environmental performance of aquaculture. pp. 1–60. doi:10.5657/FAS.2014.0001.

[mbt212369-bib-0048] Westlake, R. (1986) Large‐scale continuous production of single cell protein. Chemie Ing. Tech. 58: 934–937. doi:10.1002/cite.330581203.

[mbt212369-bib-0049] Wiebe, M.G. (2004) QuornTM Myco‐protein ‐ Overview of a successful fungal product. Mycologist 18: 17–20. doi:10.1017/S0269915X04001089.

